# A better future for children with cancer in Africa: a dream transforming into reality Dr. D Cristina Stefan- AORTIC president

**DOI:** 10.1186/s13027-019-0252-7

**Published:** 2019-11-08

**Authors:** Daniela Cristina Stefan

**Affiliations:** Director African Medical Research and Innovation Institute (AMRII), Cape Town, South Africa

## Abstract

The WHO Global Initiative for Childhood Cancer launched in 2018 will translate into an additional one million lives saved or a survival rate of at least 60% for children with cancer to be attained by 2030. This new target represents a doubling of the global cure rate for children with cancer. African children with cancer will be amongst the global group which will benefit from an improved cancer care and better outcome.

2018 will be remembered as the year when hopes for a better future for children with cancer in Africa started to transform into reality. The landmark event of the year was the launch by WHO of the Global Initiative in Childhood Cancer initiative which was supported by initial funding from St. Jude Children’s Research Hospital in the USA. St. Jude, the first WHO Collaborating Centre on childhood cancer, has committed US$ 15,000,000 to supporting implementation of the initiative. Other prominent organizations, like the American Childhood Cancer Organization and SIOP (Societe Internationale d’Oncologie Pediatrique) announced their support for the WHO Initiative

In Africa, in 2018, the real incidence or prevalence of childhood cancer is still not known. However, the overall incidence rates might be lower than reported incidence in high income countries due to the fact that almost the majority of the African population (43%) is represented by children. It means that childhood cancer is proportionately more common (4.6% of cancers in sub Saharan Africa v 0.5% in high income countries) [1].

While the survival rate of many childhood cancers surpassed 80% in resource rich countries, in low mid-income countries, where the access to care remains limited, survival rates of 20% or less are not uncommon. However, the mere estimation of the number of children newly diagnosed with cancer every year world-wide does not give any indication of the emotional, social or economic impact of the disease.

The credo of Danny Thomas, the founder of St. Jude, was that “no child should die in the dawn of life”. In the spirit of this ambitious goal, the World Health Organization Member States showed their commitment to the 2030 UN agenda for sustainable development including universal health coverage and the access of all children to a basic package of quality and palliative care services. The newly launched initiative, in September 2018, aims at supporting and encouraging governments in assessing the current capacities in cancer diagnosis and treatment with the view of improving the availability of medicines and technologies, analyzing cost and integrating childhood cancer into national strategies, social insurance and health package benefits.

The World Health Assembly Resolution 70.12 (cancer prevention and control in the context of an integrated approach) served as a foundation for the newly launched global initiative, which had as shared objective to leverage strong, committed partnerships, technical expertise to identify strategies to address the global childhood cancer burden. It is expected that, as a result of the WHO Global Initiative for Childhood Cancer, a survival rate of at least 60% for children with cancer to be attained by 2030, thereby saving an additional one million lives. This new target represents a doubling of the global cure rate for children with cancer.

“Too many children have their lives cut short by cancer, and survival rates in poor countries are scandalously lower than those in wealthy countries,” said Dr. Tedros Adhanom Ghebreyesus, WHO Director-General.

The WHO Initiative was announced right after the Third Global High-Level Meeting on Non-communicable Diseases, where dozens of heads of state and ministers from numerous countries agreed on more urgent action on noncommunicable diseases, which kill 41 million people each year. Their decision gives a renewed impetus to the achievement of the Sustainable Development Goals (SDGs), in particular SDG target 3.4 to reduce premature mortality from noncommunicable diseases by one third, by 2030.

AORTIC salutes this important WHO initiative and will support it with all its energy, to transform it into a historical turning point in the fight against childhood cancer.

We have learned and continue to learn from the past, through implementing various cancer control initiatives, that accelerated progress can be attained through concerted efforts.

## About AORTIC

AORTIC is dedicated to the facilitation of research and training as well as the provision of relevant and accurate information on the prevention, early diagnosis, treatment, and palliation of cancer, including increasing public awareness of cancer and reducing the stigma associated with it. We strive to unite the African continent in achieving its goal to reduce the cancer burden on the continent and improve outcomes of the cancer patient in Africa through collaboration with health ministries and global cancer organisations.

Addressing the cancer burden and its disproportionate impact on developing countries is an urgent task that calls for increased international partnerships and collaborations. A task that AORTIC has undertaken in order to build capacity for cancer advocacy, research and training in Africa.

The next AORTIC International Cancer conference, “Cancer in Africa: Innovation, Strategies & Implementation” will take place from 5 to 8 November 2019 in Maputo, Mozambique.

## Data Availability

(Mandatory for Biology and Medical journals)- not applicable.
